# Illusory Motion and Mislocalization of Temporally Offset Target in Apparent Motion Display

**DOI:** 10.3389/fpsyg.2013.00196

**Published:** 2013-04-19

**Authors:** Souta Hidaka, Masayoshi Nagai

**Affiliations:** ^1^Department of Psychology, Graduate School of Arts and Letters, Tohoku UniversityMiyagi, Japan; ^2^Department of Psychology, Rikkyo UniversitySaitama, Japan; ^3^National Institute of Advanced Industrial Science and Technology (AIST)Ibaraki, Japan

**Keywords:** temporal offset, illusory motion perception, perceived mislocalization, apparent motion, postdiction

## Abstract

When a visual target briefly appears in a display containing visual motion information, the perceived position of the target is mislocalized forward along its direction of motion. This phenomenon is assumed to be caused by the interaction between the transient onset signal of the target and motion information. However, while transient onset and offset signals are important for the establishment of our perceptual awareness, it has not been examined whether transient offset signals could be also effective for target mislocalization. Here, we demonstrate that shifts in perceived position occurred for a visual target containing a temporally transient offset signal in an apparent motion (AM) display. First, with horizontal AM, we found that illusory motion was perceived when a static target transiently and repeatedly blinked at a fixed position. The perceived direction of the illusory motion was in counter-phase with that of the AM stimuli. Further, we confirmed that illusory motion was frequently perceived when (1) the eccentricity of the target was larger, (2) offset duration was longer, and (3) smoother AM was perceived. Illusory motion was not perceived unless AM stimuli were presented after the offset signal, while illusory motion still occurred when the AM stimuli disappeared before the offset signal. In addition, we found that mislocalization of the target’s perceived position actually occurred in a direction opposite to AM. These findings suggest that a transient offset signal could trigger perceptual mislocalization of static visual stimuli by interacting with motion information in a postdictive manner.

## Introduction

When we focus on an object in a scene, we do not receive information solely about that object. Rather, our perceptual systems are strongly affected by the surrounding information and context around the objects we see. Not only form information (shape, texture, etc.) but also motion information influences the establishment of our perception/awareness. For example, motion information induces mislocalization of the perceived positions of objects: when a visual target briefly appears in a display containing visual motion information, the target’s perceived position is mislocalized in the forward direction of motion in both continuous motion (Whitney and Cavanagh, 2000) and apparent motion (AM; Shim and Cavanagh, 2004) displays (Flash-drag effect, FDE). In cases when observers judge the relative positions of a visual target and moving stimuli, the target is perceived as being behind the moving stimuli when they are actually aligned (Flash-lag effect, FLE; MacKay, 1958; Nijhawan, 1994; Eagleman and Sejnowski, 2000). While some explanatory hypotheses have been suggested regarding these phenomena (see Whitney, 2002 for review), a recent model indicates that motion information consisting of both spatial and temporal information plays a key role (Eagleman and Sejnowski, 2007).

In addition to motion information, temporally transient onset signal of the target could be also important for triggering mislocalization. In fact, many studies have demonstrated that transient signals contribute substantially to our perceptual awareness (e.g., Kanai and Kamitani, 2003; Kawabe et al., 2007). It could be also notable that the position of a visual stimulus is relatively uncertain when the stimulus is presented briefly (appropriately 20 ms in many cases). Based on these characteristics, the mislocalization induced by motion information has mainly been demonstrated for targets containing a transient onset signal. However, both transient onset and offset signals are assumed to be involved in the establishment of our perceptual awareness. For example, Macknik and Livingstone (1998) investigated the relationship between forward/backward masking and neural responses. They found that in a forward masking situation in which the onset of a mask temporally preceded that of the target, the mask suppressed neural responses related to the target onset signal. In contrast, masks presented after the target suppressed neural responses to the target offset signal in the backward masking situation. Perceptual awareness of the target stimuli was inhibited equally in both situations. On the basis of those findings, we could hypothesize that transient offset signals would also interact with motion information and induce mislocalization of the perceived target position.

**Figure 1:**
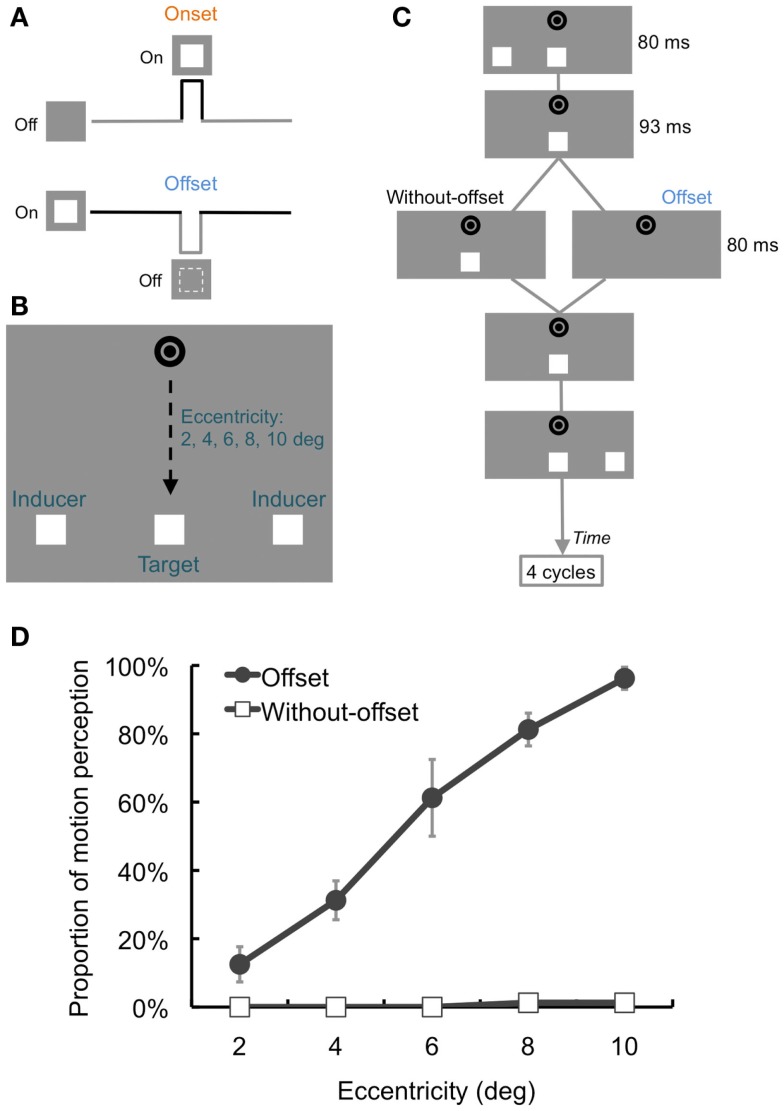
**Schematic illustrations of the stimuli, procedures, and results of Section “Experiment 1.”** **(A)** Onset and offset signals. Whereas the onset signal contains on-off transient changes, the offset signal contains opposite off-on transient ones. **(B)** Stimuli. The target stimulus was presented in-between apparent motion (AM) trajectory with 2–10° of eccentricity. **(C)** Time course of stimulus presentation. The target continuously appeared during AM in the Without-offset condition, while the target transiently disappeared for 80 ms in the Offset condition. The target position was fixed in either case. AM sequences were presented for four cycles. **(D)** Results. Proportion of illusory motion perception perceived for the target. The proportion gradually increased with the increment of the eccentricity. Error bars denote standard errors of the mean (*N* = 4).

The aim of this study was to examine whether a target containing a temporally transient offset signal could be perceptually mislocalized by motion information. In this study, AM was introduced as motion information, because the quality and direction of a motion signal is easy to manipulate by simply modifying the spatiotemporal properties of the inducers of AM (Wertheimer, 1912; Korte, 1915; Kolers, 1972). In previous literature, targets with transient onset signals were presented as brief onset-offset signal. On the contrary, the present study presented transient offset signals as brief offset-onset signal (Figure 1A). In addition to physical differences in the presentation order of the transient signals (onset-first or offset-first), the phenomenological aspects of these signals should also differ. Transient onset signals could be a cue for the sudden appearance of an object within a scene. Thus, previous studies that have adapted transient onset signals have mainly investigated the effect of motion information on the initial positional encoding process of an object. In contrast, a transient offset signal would indicate the abrupt disappearance and reappearance of an object. Therefore, by focusing on the transient offset signal, the current study could shed light on whether and how motion information could affect the re-encoding process of an object’s positional information. In this case, an object’s positional information might be compared before and after the transient offset of the object. Additionally, the target stimuli seemed to contain relatively certain positional information, because the duration of target presentation was longer than that of targets with temporally transient onset signals.

In a phenomenal observation, we found that shifts in perceived position occurred strongly for visual targets with temporally transient offset signals: illusory motion was perceived for a static target blinking at a fixed position with horizontal AM (Movie S1 in Supplementary Material). The perceived direction of illusory motion was in counter-phase with that of the AM stimuli. Our experiments further confirmed that illusory motion perception frequently occurred when (1) the eccentricity of the target was larger (Experiment 1), (2) offset duration was longer (Experiment 2), and (3) smoother AM was perceived (Experiment 3). Further, illusory motion was not perceived unless AM stimuli were presented after the offset signal, whereas it was perceived when the AM stimuli disappeared before the offset signal (Experiment 4). We further found that mislocalization of the perceived position of the target actually occurred in a direction opposite to AM (Experiments 5 and 6). These findings suggest that a transient offset signal could trigger the perceptual mislocalization of static visual stimuli by interacting with motion information in a postdictive manner.

## Experiment 1

In Experiment 1, we investigated the spatial aspect of illusory motion perception for temporally offset target stimuli presented in conjunction with AM stimuli (Movie S1 in Supplementary Material). We manipulated the vertical distances between a fixation point and the target and AM stimuli (i.e., eccentricity) and compared how frequently illusory motion perception occurred for target stimuli blinking at a fixed position.

### Material and methods

#### Participants and apparatus

Written consent was obtained from each participant before the experiments were initiated. All experiments were approved by the local ethics committee of Tohoku University. One of the authors (Souta Hidaka) and three volunteers participated in the first experiment. The volunteers were naive to the purpose of this experiment. All had normal or corrected-to-normal vision. The visual stimuli were presented on a linearized CRT display (Sony Trinitron GDM-FW900, 24″) with a resolution of 1280 × 960 pixels and a refresh rate of 75 Hz. An Apple Power Mac G4 and MATLAB (MathWorks) with the Psychophysics Toolbox (Brainard, 1997; Pelli, 1997) were used to control the experiment. The participants placed their heads on a chin rest and reported their responses using the “1” (indicating static) or “3” (indicating moving) keys on a numeric keyboard.

#### Stimuli

We presented white squares (59.98 cd/m^2^, 0.8° × 0.8°) as target stimuli and inducers of AM against a gray background (29.98 cd/m^2^; Figure 1B). The inducers and target were aligned horizontally, and the distance between the inducers was 8°. The target was presented between the inducers, so that the distance between the target and inducers was 4°. Two black rings (0.1 cd/m^2^) were presented as a fixation at the center of the display. The fixation and target were aligned vertically. The vertical distance (eccentricity) between the fixation and the target/inducers was either 2°, 4°, 6°, 8°, or 10°. The duration of the inducers was 80 ms, and the inter-stimulus interval (ISI) was 266 ms.

#### Procedure

After the presentation of the fixation circles for 500 ms, the inducers were presented as shifting from either left to right or vice versa. In each trial, four AM sequences were presented in which AM stimuli were perceived as moving back and forth horizontally. In the Without-offset condition, the target was presented continuously during AM. In contrast, the target disappeared for 93 ms after the offset of the inducers and then reappeared after 80 ms of the target’s disappearance in-between each AM sequence in the Offset condition (Figure 1C). The target was statically presented at the same fixed position in both conditions. The participants’ task was to report whether or not they perceived the target as moving. The experiment consisted of 200 trials: Target offset (2) × Eccentricity (5) × Repetition (20). These conditions were randomly introduced in each trial and were counterbalanced across participants. The initial position of the inducers (left or right) was also randomized and counterbalanced among conditions and trials.

### Results and discussion

We plotted the proportion of trials in which the target was judged as moving during the presentation of AM sequences (Figure 1D). A two-way repeated-measures analysis of variance (ANOVA) was conducted with Target offset (2) × Eccentricity (5). This analysis revealed a significant interaction between the factors [*F*(4, 12) = 35.55, *p* < 0.001]. The simple main effects of Target offset revealed that the proportion in the Offset condition was higher than that in the Without-offset condition under 4°, 6°, 8°, and 10° of eccentricity [*F*s(1, 15) > 17.17, *p*s < 0.001]. Regarding the simple main effect of Eccentricity in the Offset condition [*F*(4, 24) = 75.24, *p* < 0.001], a *post hoc* test (Tukey’s HSD) found that the proportion increased in correspondence with higher eccentricity (*p*s < 0.05).

The results suggested that the transient offset signal induced illusory motion perception of the target in an AM display. We also found that the proportion of illusory motion perception became greater with increased eccentricity. This would indicate that the target’s offset signal interacts with AM information more efficiently under larger eccentricities. This idea echoes the fact that whereas the visibility and spatial uncertainty of stimuli decreases with increased retinal eccentricity, sensitivity to motion remains constant (Koenderink et al., 1985).

## Experiment 1B

In Experiment 1, the eccentricities were manipulated for both the target and AM stimuli. In order to test whether the illusory motion perception could occur for the temporally offset target, even when the target was presented outside of the AM trajectory, we only manipulated the eccentricities of the target while those of the AM stimuli were fixed (Movie S2 in Supplementary Material).

### Methods

**Figure 2:**
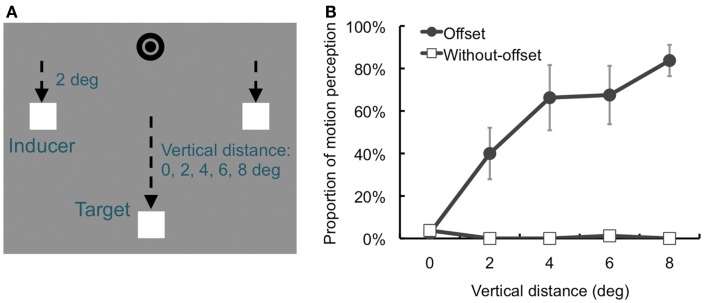
**Schematic illustrations of stimuli and results of Section “Experiment 1B.”** **(A)** Stimuli. We manipulated the vertical distance of the target as 0°, 2°, 4°, or 8° from AM stimuli, which were presented at a fixed position (2° of eccentricity from the fixation point). **(B)** Results. Proportion of illusory motion perception perceived for the target increased with the increment of the target’s eccentricity. Error bars denote standard errors of the mean (*N* = 4).

One of the authors (Souta Hidaka) and three volunteers participated in this experiment. We manipulated the vertical distance of the target as 0°, 2°, 4°, 6°, or 8° from AM stimuli, which were presented at a fixed position (2° of eccentricity from the fixation point) (Figure 2A). Except for this, the apparatus, stimulus parameters, and procedures were identical to those in Section “Experiment 1.”

### Results and discussion

Regarding the proportion of illusory motion perception (Figure 2B), a two-way repeated-measures ANOVA with Target offset (2) × Vertical distances (5) revealed a significant interaction between the factors [*F*(4, 12) = 15.29, *p* < 0.001]. The simple main effects of Target offset revealed that the proportion of motion perception in the Offset condition was higher than that in the Without-offset condition under 2°, 4°, 6°, and 8° of vertical eccentricity [*F*s(1, 15) > 9.72, *p*s < 0.001]. Regarding the simple main effect of Vertical distance in the Offset condition [*F*(4, 24) = 30.87, *p* < 0.001], *post hoc* tests (Tukey’s HSD) found that the proportions of motion perception under 4°, 6°, and 8° of vertical eccentricity were higher than those under 0° and 2° of eccentricity (*p*s < 0.05). These results indicate that illusory motion perception occurred for targets with temporally offset signals, even when the targets were located outside the trajectory of AM.

## Experiment 2

The purpose of Experiment 2 was to examine a temporal aspect of illusory motion perception of the temporally offset target. We investigated what offset duration was sufficient for the perception of illusory motion.

### Methods

One of the authors (Souta Hidaka) and three volunteers participated in this experiment. The volunteers were naive to the purpose of this experiment. All participants had normal or corrected-to-normal vision. In this experiment, the ISI of the inducers was 267 ms. The offset duration was either 0 (Without-offset), 27, 53, 80, 107, or 133 ms. The eccentricities of the target and AM stimuli were fixed at 8°. The main session consisted of 120 trials: Offset duration (6) × Repetition (20). The order of the conditions was randomized and counterbalanced across trials and participants. Except for these differences, the apparatus, stimulus parameters, and procedures were identical to those in Section “Experiment 1.”

### Results and discussion

**Figure 3:**
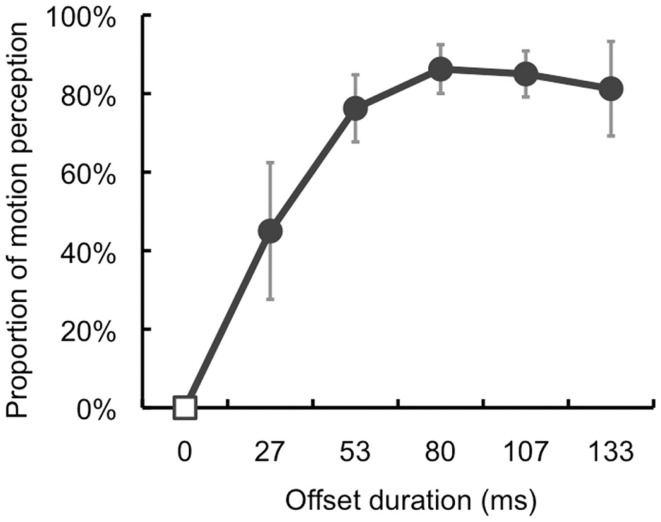
**Results of Section “Experiment 2.”** The proportion of illusory motion perception increased with longer offset duration. Error bars denote standard errors of the mean (*N* = 4).

Regarding the proportion of illusory motion perception (Figure 3), a one-way repeated-measures ANOVA revealed a significant main effect of Offset duration [*F*(5, 15) = 16.69, *p* < 0.001]. The *post hoc* test (*p* < 0.05) found that the proportion of motion perception at 27 ms offset was higher than that with 0 ms offset. Further, the proportions at 53, 80, 107, and 133 ms offset duration were higher than those in the other conditions.

The results showed that the target’s temporally offset signal induced illusory motion perception more frequently with longer offset durations and that 53 ms of offset duration was sufficient to trigger illusory motion perception reliably.

## Experiment 3

The results of Section “Experiments 1 and 2” clearly showed that illusory motion perception occurred for targets with temporally offset signals in an AM display. Given that illusory motion perception occurred due to the interaction between motion information from AM stimuli and the target’s temporally offset signal, we predicted that illusory motion perception would be directly related to AM perception. The perceived quality (smoothness or goodness) of AM could be experimentally altered by changes in ISI under a fixed distance between the target and inducers (Korte, 1915; Kolers, 1972). Thus, we examined the effects of the perceived motion quality of AM on illusory motion perception by manipulating the ISI of the inducers.

### Methods

One of the authors (Souta Hidaka) and three volunteers participated in this experiment. The volunteers were naive to the purpose of this experiment. All the participants had normal or corrected-to-normal vision. The ISI of the inducers was either 134, 186, 294, 506, 934, or 1786 ms. The eccentricities of the target and AM stimuli were fixed at 8°. Only the Offset condition was presented. First, the participants completed a motion-judgment session wherein they were asked to judge whether or not the target was perceived as moving. This session consisted of 120 trials: ISI (6) × Repetition (20). In the subsequent motion-quality-judgment session, we asked the participants to judge perceived motion quality (smoothness, goodness, etc.) of AM stimuli by using a five-point scale [from 1 (bad) to 5 (good)]. This session consisted of 60 trials: ISI (6) × Repetition (10). The conditions were randomly assigned and counterbalanced among the trials and participants. Except for these differences, the apparatus, stimulus parameters, and procedures were identical to those in Section “Experiment 1.”

### Results and discussion

**Figure 4:**
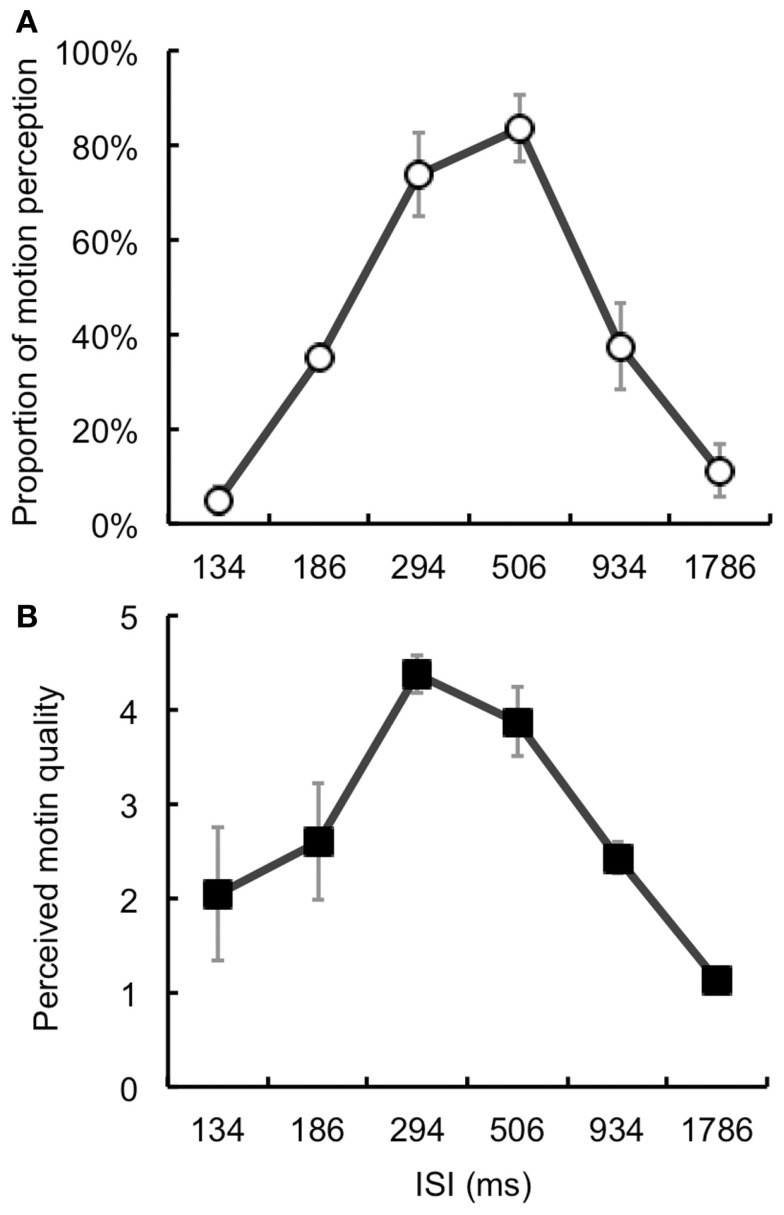
**Results of Section “Experiment 3.”** **(A)** Proportion of illusory motion perception for the target. **(B)** Perceived motion quality. The values peaked with ISIs of 294 and 506 ms. We also confirmed a significant positive correlation between these values (*r* = 0.83). Error bars denote standard errors of the mean (*N* = 4).

With regard to the obtained proportion of motion perception (Figure 4A), a one-way repeated-measures ANOVA found a significant main effect of ISI [*F*(5, 15) = 17.87, *p* < 0.001]. The *post hoc* tests (*p* < 0.05) revealed that the proportions with 294 and 506 ms ISI were higher than those for the other ISI values. We also calculated the correlation coefficient (Spearman’s *r*) between the proportion of motion perception and perceived motion quality (Figure 4B). The estimated correlation was *r* = 0.83, which was statistically significant (*p* < 0.05, one-tailed).

The results showed that illusory motion perception selectively occurred with particular ISI values. Moreover, this tendency was highly related to the perceived motion quality of AM. Thus, we could consider illusory motion perception of the temporally offset target to be directly related to motion information.

## Experiment 4

The results of Section “Experiments 1 and 3” suggested that the interaction between the target’s temporally offset signal and AM information could be an important factor of illusory motion perception. Since the previous experiments repeatedly presented AM sequences and temporally offset targets, it was uncertain whether the presentation of AM information before or after the offset signal – or both – primarily contributed to the perception of illusory motion. To test this, we introduced the absence of inducers before or after the presentation of the offset signal. We could predict that if AM information presented before the offset signal plays a key role, then illusory motion perception would not occur unless the inducer was presented before target offset. On the other hand, if AM information presented after target offset is critical, then illusory motion would not be perceived unless the inducer was presented after the offset.

### Methods

**Figure 5:**
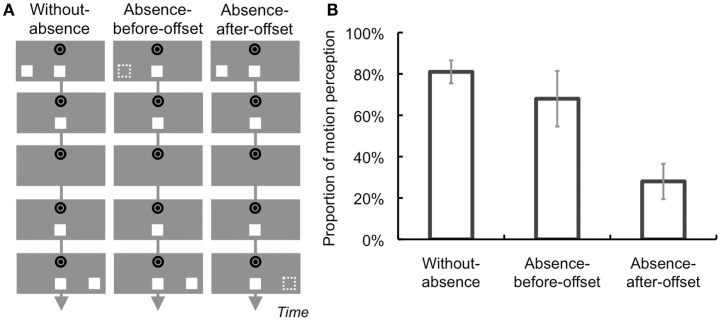
**Schematic illustrations of conditions and results of Section “Experiment 4.”** **(A)** Conditions. Apparent motion (AM) inducers were continuously presented at the last AM sequence in the Without-absence condition. In contrast, the inducer was not presented before or after target offset in the Absence-before-offset and Absence-after-offset conditions, respectively. **(B)** Results. The proportion of illusory motion perception of the target was smaller in the Absence-after-offset condition than in the other conditions. Error bars denote standard errors of the mean (*N* = 4).

One of the authors (Souta Hidaka) and three volunteers participated in this experiment. The volunteers were naive to the purpose of this experiment. All the participants had normal or corrected-to-normal vision. In each trial, four AM sequences were presented. In the Without-absence condition, the inducers were continuously presented in all the sequences. However, in the Absence-before-offset condition, the inducer was not presented before the target’s temporal offset during the last AM sequence. On the contrary, in the Absence-after-offset condition, the inducer did not appear after target’s temporal offset during the last AM sequence (Figure 5A). The eccentricities of the target and AM stimuli were fixed at 8°. The participants completed 60 trials: Condition (3) × Repetition (20). The conditions were randomly assigned and counterbalanced across trials and participants. Except for these differences, the apparatus, stimulus parameters, and procedures were identical to those in Section “Experiment 1.”

### Results and discussion

A one-way repeated-measures ANOVA found a significant main effect of Condition [*F*(2, 8) = 8.43, *p* < 0.05; Figure 5B]. The *post hoc* tests (*p* < 0.05) revealed that the proportion of perceived motion in the Absence-after-offset condition was lower than that in the other conditions.

The results showed that the proportion of illusory motion perception was reduced when the inducer was not presented after target’s temporal offset. This would indicate that AM information presented after target’s offset mainly contributes to illusory motion perception in a postdictive manner. A reliable amount of illusory motion perception occurred in the Absence-before-offset condition, although AM information was not explicitly presented during the last sequence (the inducers were presented twice at the same position.) This might be because, in addition to that the repeated presentation of AM sequences might introduce AM information implicitly and predictively, AM perception might also occur between the target (at the center of the display) and the inducer when it is presented after the offset.

## Experiment 5

In the previous experiments, we demonstrated that a target with a temporally offset signal was perceived as moving within an AM display, even though the target was actually presented at a fixed position. The underlying mechanism of this effect could be that AM information induced perceived shifts of the target’s position (e.g., Whitney and Cavanagh, 2000; Shim and Cavanagh, 2004). To confirm this possibility, in Experiment 5, we measured the magnitude of mislocalization of the temporally offset target in an AM display.

### Methods

**Figure 6:**
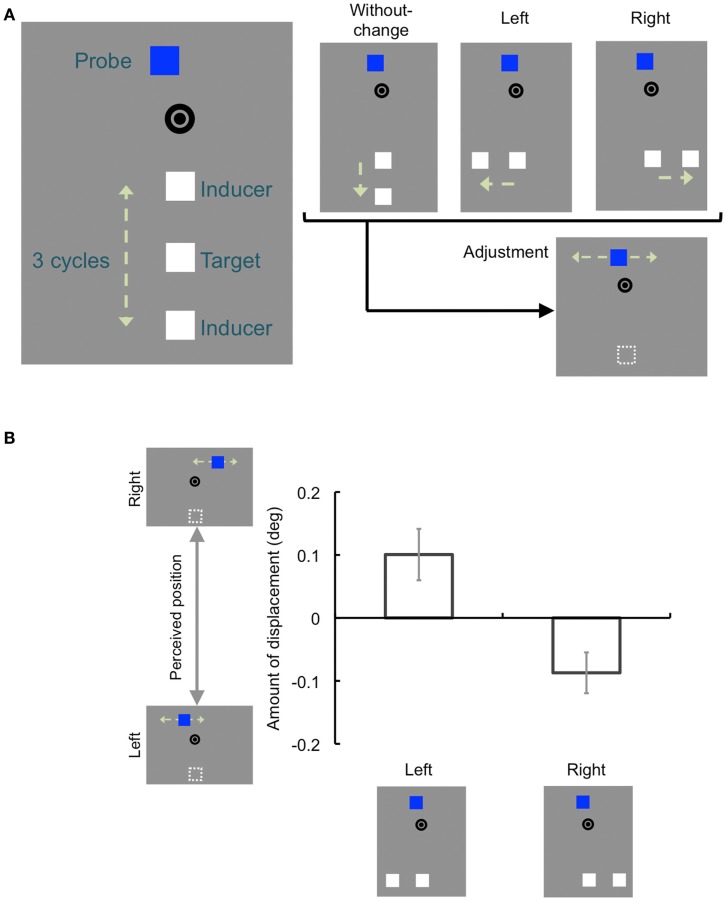
**Schematic illustrations of the stimuli, conditions, and results of Section “Experiment 5.”** **(A)** Stimuli and conditions. Vertical apparent motion (AM) sequences were presented for the first three cycles. A probe square was also presented at a randomly assigned horizontal position (±0.8° ) above a fixation point during AM. In the subsequent, final AM sequence, while the horizontal position of AM stimulus was constant in the Without-change condition, the position shifted toward the left or right in the Left and Right conditions, respectively. Then, participants were asked to indicate the final perceived position of the target by adjusting the probe position. **(B)** Results. The adjustments in the Left and Right conditions were normalized against those in the Without-change condition. The adjustments shifted in a direction opposite to that of AM. Error bars denote standard errors of the mean (*N* = 8).

One of the authors (Souta Hidaka) and seven volunteers participated in this experiment. The volunteers were naive to the purpose of this experiment. All the participants had normal or corrected-to-normal vision. We presented a blue probe square (17.37 cd/m^2^, 0.8°  × 0.8° ) at 6.7° above the fixation point. The horizontal position of the probe was randomly selected within ±0.8° around the center of the display in each trial. The target was presented 8° below the fixation point. The inducers were presented at 4° above and below the target, while their horizontal positions were aligned with the target (Figure 6A). We presented three AM sequences in the vertical direction. Then, for the subsequent, final (4th) sequence, the final position of the inducer was moved to a location 4° either to the left (Left condition) or right (Right condition) of the target. The vertical positions of the inducers were aligned with that of the target. A condition in which the positions of the inducers were not changed was also introduced (Without-change condition). Only the offset condition was presented, so that the target always transiently disappeared between presentations of the inducers. The participants were asked to adjust the horizontal position of the probe to a location consistent with the perceived final location of the target while focusing on the fixation point. The participants completed 60 trials: Condition (3) × Repetition (20). The order of conditions was randomized and counterbalanced across trials and participants. Except for these differences, the apparatus, stimulus parameters, and procedures were identical to those in Section “Experiment 1.”

### Results and discussion

We normalized each participant’s data by subtracting the adjustments made in the Without-change condition from those in the Left and Right conditions (Figure 6B). Then, we conducted a two-tailed, paired *t* test, which revealed significant difference between the Left and Right conditions [*t*(7) = 2.98, *p* < 0.05]: the adjustments shifted to the right in the Left condition and to the left in the Right condition.

The results showed that the shifts in perceived position actually occurred for the temporally offset targets. In addition, although the inducers’ positions in the last display were randomly assigned across conditions and trials, the perceived shifts were consistently against the direction of AM. Consistent with the results of the previous experiments, these results indicate that perceptual mislocalization of the target occurred postdictively and that the direction of the perceptual shift was opposite to the direction of AM.

## Experiment 6

In the previous experiments, the AM sequences and target’s offset signals were repeatedly presented in a few cycles. In contrast, studies have demonstrated that perceived mislocalization for the target with a temporally onset signal could occur even with a single presentation of the onset signal and AM sequence (e.g., Eagleman and Sejnowski, 2007). Thus, in Experiment 6, we tested whether the temporally offset target could be perceptually mislocalized when the target offset signal and AM sequence were presented only once. As in Section “Experiment 5,” we introduced a situation where the AM direction was unpredictable and determined only after the target offset was presented.

### Methods

**Figure 7:**
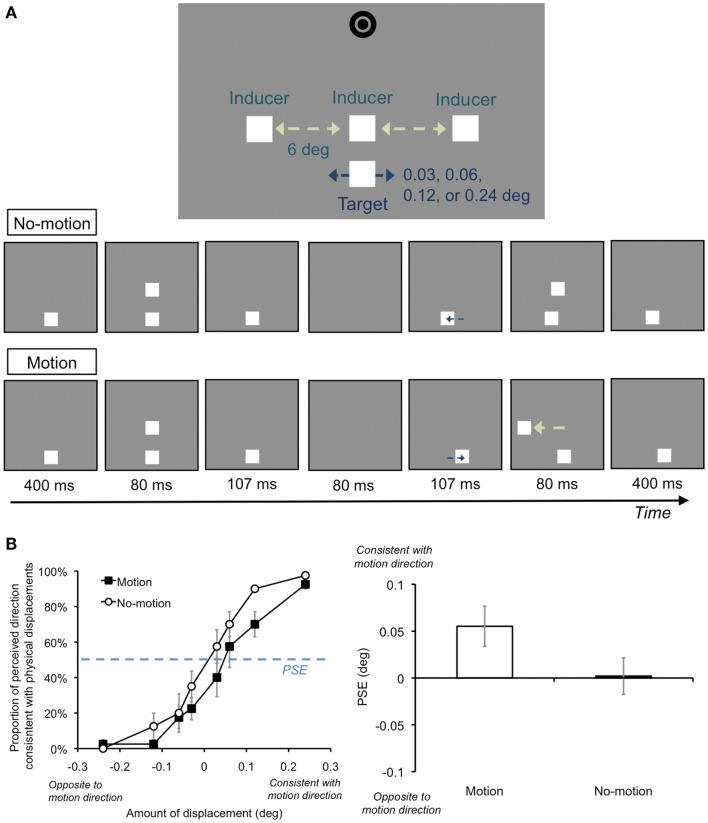
**Schematic illustrations of the stimuli, conditions, and results of Section “Experiment 6.”** **(A)** Stimuli and conditions. We presented the target and one of the inducers at the center of the display. In each trial, after 400 ms of the target presentation, the inducer was presented for 80 ms. Next, the target was temporally offset for 80 ms. The target subsequently reappeared, and its horizontal position was displaced either 0.03°, 0.06°, 0.12°, or 0.24° in the left or right direction. An inducer was then presented. While the inducer was presented at the same position as the first inducer in the No-motion condition, the inducer’s position was shifted toward the left or right in the Motion condition. **(B)** We plotted the proportion at which the perceived direction of the target was consistent with the physical displacements in each motion condition. Positive values of the target’s displacements indicate that the displacements were consistent with the AM direction in the Motion condition. We then estimated the point of subjective equality. The PSE shifted in the direction consistent with the AM so that the target displacements tended to be perceived to be opposite the AM direction in the Motion condition. Error bars denote standard errors of the mean (*N* = 4).

One of the authors (Souta Hidaka) and three volunteers participated in this experiment. We presented the target and one of the inducers at the center of the display, 8° and 6° below the fixation points, respectively (cf. Eagleman and Sejnowski, 2007) (Figure 7A). These stimuli were horizontally aligned. In order to quantify the amount of perceived mislocalization for the target, we adopted a nulling procedure. In each trial, after 400 ms of the target presentation, the inducer was presented for 80 ms. Next, the target was temporally offset for 80 ms. The target subsequently reappeared, and its horizontal position was displaced either 0.03°, 0.06°, 0.12°, or 0.24° in the left or right direction. An inducer was then presented. While the inducer was presented at the same position as the first inducer in the No-motion condition, the inducer’s position was shifted 6° toward the left or right in the Motion condition. Participants were asked to judge the perceived direction of the target’s displacement (left or right). Participants completed 160 trials: Motion (2) × Target displacements (8) × Repetitions (10). The order of the conditions was randomized and counterbalanced among trials and participants. Further, the amount of the target’s displacements and the direction of the target’s displacements and AM were randomly introduced in each trial and counterbalanced among the conditions. Except for these differences, the apparatus, stimulus parameters, and procedures were identical to those in Section “Experiment 1.”

### Results and discussion

For each participant, we plotted the proportion at which the perceived direction of the target was consistent with the physical displacements in each motion condition (Figure 7B). Positive values of the target’s displacements indicate that the displacements were consistent with the AM direction in the Motion condition. We then estimated the point of subjective equality (PSE) by fitting a cumulative Gaussian distribution function to each participant’s data by using a maximum likelihood method. A two-tailed paired *t* test revealed a significant difference between the Motion and No-motion conditions [*t*(3) = 9.70, *p* < 0.005]. Since the PSE shifted in the direction consistent with the AM, the target displacements tended to be perceived to be opposite the AM direction in the Motion condition. These results indicate that a reliable amount of perceptual displacements could postdictively occur for the temporally offset target, even when the target offset signal and AM sequence were presented only once.

## General Discussion

It has been reported that the perceived position of a target with a transient onset signal is mislocalized in the forward direction with respect to nearby motion information. The aim of the present study was to investigate whether a transient offset signal would also induce mislocalization of the perceived position of the target. Phenomenological observation revealed that illusory motion was perceived for the target blinking at a fixed position in counter to the direction of horizontal AM stimuli (Movie S1 in Supplementary Material). Illusory motion was frequently perceived when (1) the eccentricity of the target was larger (Experiment 1), (2) offset duration was longer (Experiment 2), and (3) smoother AM was perceived (Experiment 3). Further, illusory motion perception did not occur when AM stimuli did not appear after the target’s offset signal (Experiment 4). We further found that mislocalization of the target’s perceived position actually occurred in a direction opposite to AM (Experiments 5 and 6). These findings suggest that a transient offset signal could trigger the perceptual mislocalization of static visual stimuli by interacting with motion information in a postdictive manner.

Eye movements induced by AM stimuli might contribute to the perception of illusory motion and target mislocalization. However, we found that illusory motion perception was modulated by changes in inducers’ ISI which was strongly related to the perceived quality of AM, although eye movements could occur irrespective of changes in ISI (Experiment 3). In addition, perceived mislocalization was consistently observed in the situation where the final location of AM stimuli was changed randomly in the last display (Experiment 5). Further, we observationally confirmed that illusory motion could also occur in the vertical direction (the direction in which eye movements are less effective; the first three AM sequences in Experiment 5). These findings would thus indicate that eye movements were not a decisive factor in the current study.

The involvement of attentional shifts might be also considered. In fact, it has been reported that shifts in attentional location induced perceived mislocalization of briefly presented targets in the direction opposite to the attentional shifts (attentional repulsion effect: Suzuki and Cavanagh, 1997). However, the phenomenological aspects of that finding could differ from those of our current ones. In the study by Suzuki and Cavanagh (1997), attentional cues were always presented before target’s onset, and AM information presented just after target’s onset did not modulate the occurrence of the effect. On the other hand, we demonstrated that AM stimuli presented after the target’s offset signal dominantly contributed to mislocalization. Another study also reported that attentional shifts induced after the onset of a target triggered perceived mislocalization of the target (Ono and Watanabe, 2011). In that case, however, the observed mislocalization was always in the direction of the attentional shifts (attentional attraction effect). While some studies have reported attentional modulation of the FDE (Shim and Cavanagh, 2005; Tse et al., 2011), attention might have only modulatory effects that help the observer to selectively track one of two competitive sources of motion information. Based on these facts, we could assume that the involvement of attentional shift/modulation would not fully explain our current findings.

**Figure 8:**
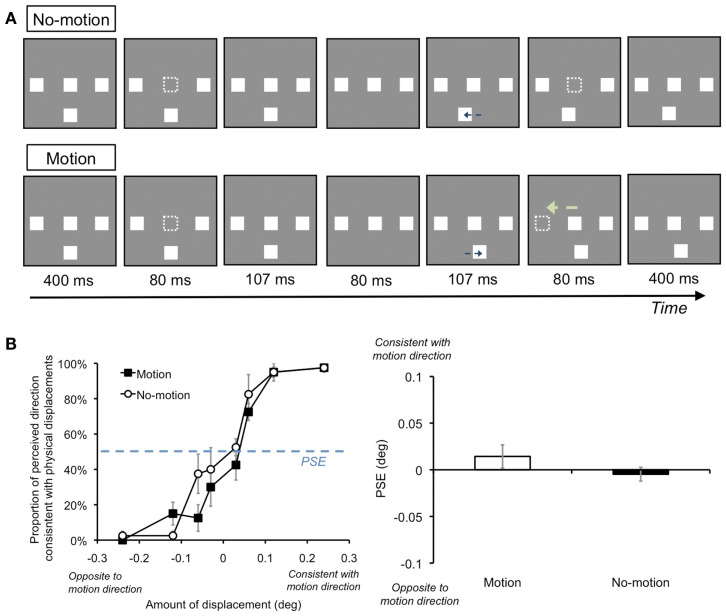
**Schematic illustrations of stimuli and results when the temporal characteristics of AM stimuli were modified for shadow motion perception (Experiment A1)**. **(A)** Stimuli. We presented AM stimuli as transiently offset so that their temporal characteristics were opposite to those in Section “Experiment 6.” During each trial, the AM stimuli were initially presented and disappeared for 80 ms at each timing point. Except for these differences, the apparatus, stimulus parameters, and procedures were identical to those in Section “Experiment 6.” **(B)** Results. We plotted the proportion at which the perceived direction of the target was consistent with the possible shadow motion direction in each motion condition. Regarding the estimated point of subjective equality (PSE), a two-tailed paired *t* test found that differences in PSEs between the Motion and No-motion conditions was not significant [*t*(3) = 1.13, *n.s*.]. These results indicate that perceptual displacements could well occur with stimulus motion of AM stimuli in our current experimental situations. Error bars denote standard errors of the mean (*N* = 4).

Thus, we could consider that mislocalization of targets containing transient offset signal occurs due to the interaction between the transient offset signal and AM information. Since illusory motion perception and perceived mislocalization for the target could occur both along (Experiments 1–5) and outside the AM trajectory (Experiments 1B and 6), the target’s offset signal could explicitly and implicitly interact with AM information. Some possible underlying mechanisms could be considered. For example, one may assume involvement of the “shadow motion” phenomenon (also called “pure phi” or “omega” motion; Saucer, 1953; Zeeman and Roelofs, 1953; Tyler, 1973; Sigman and Rock, 1974; Gellatly and Blurton, 1995; Ekroll et al., 2008). Typically, in this phenomenon, when two white squares on a black background (horizontally apart from each other) are alternately turned on and off, depending on particular temporal properties, AM for the white squares (“stimulus motion”) is not perceived. Rather, the blank (offset) points of the squares are perceived as a black “shadow” that appears to move counter to the onset of the white squares. This phenomenon might suggest that in our experimental situation, shadow motion was perceived for the AM stimuli, and the temporally offset target was perceptually grouped together with the AM stimuli. Consequently, illusory motion was perceived for the target counter to the direction of the horizontal AM stimuli. A notable point is that the temporal characteristics of our AM stimuli would be optimal for stimulus motion. Indeed, the results of Section “Experiment 3” showed that the perceived quality of stimulus motion induced by the AM stimuli became higher at the particular ISIs when the participants were asked to directly judge the motion. Illusory motion perception occurred most frequently at these ISIs. In addition, Ekroll et al. (2008) showed that optimal temporal properties were contradictory between stimulus and shadow motion perception. In other words, while stimulus motion prefers that AM stimuli contain transient onset signals, shadow motion prefers AM stimuli with transient offset signals. Thus, these motion perceptions should occur exclusively. Actually, as shown in our demonstration movie, we may not perceive shadow motion but may mainly perceive stimulus motion with our stimuli (see Movie S1 in Supplementary Material). We also create a demonstration in which shadow motion, instead of stimulus motion, may be dominantly perceived (Movie S3 in Supplementary Material). Whereas illusory motion perception may be vividly perceived in the former case, illusory motion may not appear or unreliably occur in the latter case. Moreover, an additional experiment (Experiment A1) found that perceived mislocalization for the target did not occur when we modified the spatiotemporal characteristics of AM stimuli in Section “Experiment 6” so that shadow motion could be perceived (Figure 8). These findings would suggest that illusory motion perception and perceived mislocalization for the target could be well observed with stimulus motion of AM stimuli. However, we should also note that our current manipulation might not be appropriate for shadow motion perception. Thus, investigations should be performed in future studies by using optimal spatiotemporal characteristics for shadow motion perception.

Furthermore, it could be notable that the existence of shadow motion mechanisms would indicate that the target’s temporally transient offset signal could potentially serve as a motion cue by itself. This would suggest another possible underlying mechanism. For instance, the involvement of the onset repulsion effect (ORE) may be considered. In this phenomenon, the onset position of a moving target tends to shift backwards along motion trajectory (Thornton, 2002). In fact, the data obtained in the Absence-before-offset condition of Section “Experiment 4” seemed to suggest that AM was perceived between the reappearing target and the subsequent AM stimuli so that the target’s position is misperceived in a backward direction, as in the ORE. Thus far, ORE has been mainly reported in a situation where the temporally onset target is presented along an AM trajectory. However, we also reported that illusory motion perception and perceived mislocalization occurred even when the target was presented outside of the AM trajectory (Experiments 1B and 6: see also Movie S2 in Supplementary Material). It may be interesting to consider that ORE could occur for the target with temporally offset signal outside the AM trajectory. Involvement of the mechanism related to FDE may also be assumed. FDE is a phenomenon whereby a visual target with a transient onset signal is mislocalized in the forward direction of a nearby motion signal (Whitney and Cavanagh, 2000; Shim and Cavanagh, 2004). There seems to be a basic phenomenological distinction: whereas forward displacements are observed in FDE, backward mislocalization consistently appeared in this study. However, it may be likely that AM information could induce forward perceived mislocalization of “shadow” element of the target. This may then result in the backward mislocalization of “stimulus” element of the target.

As perceptual mislocalization occurred in the backward motion direction and in a postdictive manner, the findings reported here may be also related to FLE. FLE is reported to occur such that a target with a transient onset signal is perceived at a backward position relative to the moving stimulus, although they are physically aligned (MacKay, 1958; Nijhawan, 1994; Eagleman and Sejnowski, 2000). The mechanism of FLE is considered to be that the target’s transient onset signal resets the spatiotemporal integration process of the nearby motion signal. Motion information presented after the target’s onset signal would be then reintegrated within a particular temporal window. This would result in motion bias: the position of the moving object is perceived as displaced toward motion direction relative to the target (Eagleman and Sejnowski, 2000, 2007). Accordingly, in the current study, the transient offset signal could also reset the process of spatiotemporal integration of motion information. In the subsequent stage, the motion signal would be reintegrated within a particular timeframe and resulting motion bias would occur, such that the target was perceptually localized relatively behind the motion signal. In addition, since the transient offset signal might phenomenologically indicate the abrupt disappearance and reappearance of an object, we may assume that the transient offset signal would also induce the reset and re-encoding of the target’s positional information: an object’s positional information might be compared before and after the transient offset. A perceptual displacement signal of the target induced by motion biasing might be attributed to the comparison process between the previous and subsequent target positions. Consequently, the target’s position after the offset may be consistently perceived as a backward position relative to the position before the offset in a postdictive manner.

Since these ideas are speculative at this stage, further investigations as to the underlying mechanisms of offset-induced mislocalization, including its postdictive aspects, should be performed in the near future. However, the current findings clearly demonstrate two novel phenomenological aspects of perceptual mislocalization of temporally offset targets. The first is that, contrary to the target containing a transient onset signal, the illusory motion and perceptual mislocalization for the target with temporally offset signal consistently occurs opposite the direction of AM information. The second aspect is that illusory motion perception and perceived mislocalization are observed for the target’s absolute position, whereas previous literature has mainly reported that the temporally onset target’s position is perceptually misaligned relative to nearby reference stimuli (Whitney and Cavanagh, 2000; Shim and Cavanagh, 2004) or nearby motion signals (Eagleman and Sejnowski, 2007; Shi and de’Sperati, 2008). In fact, the mislocalization occurred strongly for temporally offset targets as they were perceived as moving back and forth, even though the targets contained relatively certain positional information (presented for approximately 180 ms) compared with targets containing transient onset signals (which were typically presented for approximately 20 ms) (Experiments 1–4). Moreover, the perceptual displacements in a backward direction consistently occurred even when the participants judged the target’s position itself (Experiments 5 and 6). Therefore, temporally offset signal would have phenomenological aspects or functions different from those of temporally onset signal in our perceptual systems.

## Conflict of Interest Statement

The authors declare that the research was conducted in the absence of any commercial or financial relationships that could be construed as a potential conflict of interest.

## Supplementary Material

The Supplementary Material for this article can be found online at: http://www.frontiersin.org/Consciousness_Research/10.3389/fpsyg.2013.00196/abstract

Supplementary Movie S1 **The basic phenomenon of illusory motion perception for a temporally offset target in an apparent motion (AM) display**. Please see a white square blinking at a lower position while fixating on the black rings. Whereas the square will be perceived as static without AM at the first sequence, the square will then appear to move to the left or right when AM is presented. The direction of illusory motion of the target will be in counter-phase with that of AM.

Supplementary Movie S2 **Demonstration of the situation where the target is not presented along an apparent motion trajectory**. This movie demonstrates the case where the vertical distance is well separated between the AM stimuli and target; the relative distance between them corresponds to 6  of the vertical distance. We confirmed that illusory motion perception reliably occurred even in this situation.

Supplementary Movie S3 **Demonstration of the situation where the shadow motion can be perceived**. We may notice that illusory motion perception does not appear or unreliably occurs contrary to when the stimulus motion can be perceived (Movie S1).
